# Modifying Antibiotic Activity of Synthetic Thiadiazine Analogs Against MDR Bacteria and ADMET Analysis

**DOI:** 10.1002/open.202500260

**Published:** 2025-10-24

**Authors:** João Arthur de Oliveira Borges, Priscilla Ramos Freitas, Isaac Moura Araújo, Ray Silva de Almeida, Igor José dos Santos Nascimento, João Xavier de Araújo‐Júnior, Edeildo Ferreira da Silva‐Júnior, Thiago Mendonça de Aquino, Francisco Jaime Bezerra Mendonça Junior, Emmanuel Silva Marinho, Hélcio Silva dos Santos, Radosław Kowalski, Grażyna Kowalska, Henrique Douglas Melo Coutinho, Saulo Relison Tintino, Ana Carolina Justino de Araújo

**Affiliations:** ^1^ Laboratory of Microbiology and Molecular Biology‐LMBM Regional University of Cariri Crato CE 63105‐000 Brazil; ^2^ Laboratory of Medicinal Chemistry Institute of Pharmaceutical Sciences Federal University of Alagoas Maceió AL 57072‐900 Brazil; ^3^ Biological and Molecular Chemistry Research Group Institute of Chemistry and Biotechnology Federal University of Alagoas Maceió AL 57072‐900 Brazil; ^4^ Laboratory of Synthesis and Research in Medicinal Chemistry Research Group on Therapeutic Strategies—GPET Institute of Chemistry and Biotechnology Federal University of Alagoas Maceió AL 57072–900 Brazil; ^5^ Department of Biological Science, Laboratory of Synthesis and Drug Delivery‐LSVM State University of Paraiba João Pessoa 58071‐160 PB Brazil; ^6^ Laboratory of Chemistry of Natural and Synthetic Product State University of Ceara´, UECE Fortaleza Ceara´ 60714‐903 Brazil; ^7^ University of Life Sciences in Lublin 20‐950 Lublin Poland

**Keywords:** bacterial resistance, biological interactions, macromolecules, *Pseudomonas aeruginosa*, *Staphylococcus aureus*

## Abstract

The indiscriminate use of antibiotics has led to the selection of resistant bacterial strains, significantly reducing the effectiveness of conventional treatments. In this context, thiadiazines have emerged as promising agents due to their antioxidant and antibacterial properties. This article aims to evaluate the antibacterial potential of synthetic thiadiazine analogs against selected bacterial strains. The synthesized compounds are purified using high‐performance liquid chromatography, and absorption, distribution, metabolism, excretion, and toxicity analyses are performed, including interaction profiling with over 370,000 bioactive compounds. The bacterial strains *Staphylococcus aureus* 10 and *Pseudomonas aeruginosa* 24 are used as test organisms. When combined with standard antibiotics, thiadiazine compounds significantly reduced the minimum inhibitory concentration. However, some analogs exhibited antagonistic effects, particularly against gentamicin and erythromycin. Direct antibacterial activity is limited, with compounds IJ26 and IJ28 showing the most notable effects. These findings suggest that thiadiazine analogs may potentiate antibiotic activity, although further studies are needed to fully understand their biological interactions and mechanisms of action.

## Graphical abstract

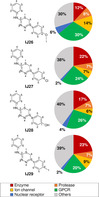

## 1 Introduction

Since Alexander Fleming's discovery of antibiotics, they have been widely used in medical practice, playing a crucial role in the fight against microorganisms. However, the extensive use of these medications has led to a growing increase in antibiotic resistance. This phenomenon represents a global threat to both human and animal health, as many bacterial species are acquiring varying levels of resistance to antibiotics.^[^ ^1^ ^]^

*Staphylococcus aureus* is a well‐known pathogenic bacterium, responsible for infections such as boils, folliculitis, carbuncles, and abscesses. It is also one of the main causes of pneumonia and other respiratory tract infections. Infections caused by S. aureus are particularly alarming due to the bacterium's ability to rapidly acquire resistance to antibiotics. Among the resistant strains, methicillin‐resistant *S. aureus* is clinically significant, as its infections are associated with greater morbidity and mortality compared to those caused by methicillin‐sensitive *S. aureus*.^[^ ^2^ ^]^

*Pseudomonas aeruginosa* is an opportunistic, Gram‐negative, nonspore‐forming bacterium found in both biotic and abiotic environments. *P. aeruginosa* can infect plants and animals, including humans, and is primarily associated with surgical site, pulmonary, and nosocomial infections. Some strains exhibit resistance to a wide range of antimicrobial agents, from carbapenems to cephalosporins. These resistance mechanisms are intrinsic, acquired, and adaptive.^[^ ^3^ ^,^ ^4^ ^]^

The excessive or inappropriate use of antibiotics can accelerate the emergence and spread of resistant bacteria, sometimes resulting in antibiotic misuse or overuse. A notable example is penicillin, where bacteria exposed to sublethal concentrations of the antibiotic develop the ability to produce β‐lactamase enzymes. These enzymes can break the amide bond of the β‐lactam ring, rendering the antibiotic ineffective.^[^ ^5^ ^]^

As an alternative approach to combating bacterial resistance, thiadiazine derivatives have been studied for various pharmacological applications. These include use as anesthetics, cardiovascular and hypometabolic agents, and for the treatment or prevention of anemia, bone growth deficiencies, tumors, and acquired immunodeficiency syndrome. Thiadiazines are also being explored in the management of heart failure, asthma, and allergies. Furthermore, they have applications in agriculture as herbicides, fungicides, insecticides, pesticides, and plant growth regulators.^[^ ^6^ ^]^

Given this context, the aim of this work is to evaluate the action of thiadiazine analogs as potentiating‐antibiotic agentes against multidrug‐resistant (MDR) bacteria. The continued development and investigation of thiadiazine synthesis are essential for validating their therapeutic potential, thereby contributing to the development of new strategies to combat antibiotic resistance.

## 2 Results

### 2.1 Chemistry

The synthesis of thiadiazine analogs was achieved through the introduction of ions into the second free radical. As shown in Supporting Information 1, the synthetic methodology used for obtaining the thiadiazine derivatives involved two main steps. Initially, N‐substituted thiosemicarbazides (1–4) were prepared by condensation of hydrazine with isothiocyanates. The spectroscopic data were consistent with previously reported literature.^[^ ^7^, ^8^, ^9^ ^–^ ^10^ ^]^ Subsequently, the final thiadiazine compounds were synthesized in a single‐step reaction by treating the N‐substituted thiosemicarbazides (1–4) with substituted phenylacetyl bromides in either methanol or acetonitrile.^[^ ^11^ ^]^ The structures of the synthesized compounds were confirmed by _1_H and _13_C NMR spectroscopy.

### 2.2 Antibacterial Activity

The minimum inhibitory concentration (MIC) method was used to determine the lowest concentration of each compound capable of inhibiting bacterial growth. When tested directly against *P. aeruginosa* and *S. aureus*, the compounds exhibited MIC values of 1024 μg mL^–^^1^, except for compound IJ28, which showed an MIC of 512 μg mL^–1^ against *S. aureus*.

It is important to note that, although the present study refers to the modulation of antibiotic activity, no formal synergy assays (such as checkerboard or fractioned inhibitory concentration index) were performed. Therefore, the results should be interpreted as indicative of potential modulatory effects (**Table** **1**).

**Table 1:** Minimum inhibitory concentration (MIC, in µg mL^–1^) values of thidiazine‐derived compounds tested against *S. aureus* and *P. aeruginosa* strains.

MIC							
Compounds	*Staphylococcus aureus* 10			Compounds	*Pseudomonas aeruginosa* 24		
IJ26	1024	1024	1024	IJ26	1024	1024	1024
IJ27	1024	1024	1024	IJ27	1024	1024	1024
IJ28	1024	1024	1024	IJ28	512	512	512
IJ28	1024	1024	1024	IJ28	1024	1024	1024

Only two MDR bacterial strains were tested, *S. aureus* (Gram‐positive) and *P. aeruginosa* (Gram‐negative), to represent the main bacterial profiles. This choice allows an initial assessment of the compounds’ activity across different types of bacteria. Future studies should include additional strains to broaden the generalizability of the findings and validate the results obtained (**Table** **2**).

**Table 2:** Values (µg mL^−^^1^) and percentual reduction of compounds 26–29 relative to reference antibiotics against *S. aureus* (SA) and *P. aeruginosa* (PA).

Modification of antibiotic activity							
Bacteria	Compounds	Gentamicin (8)	Reduction [%]	Erythromycin (0,5)	Reduction [%]	Norfloxacin (203,18)	Reduction [%]
**SA**	**IJ26**	10.07	−259	64	−12600	10.07	95.0
	**IJ27**	10.07	−25.9	64	−12600	10.07	95.0
	**IJ28**	6.34	20.8	128	−25500	6.34	96.9
	**IJ29**	16	−100	203.18	−40536	64	68.5
Bacteria	Compounds	Gentamicin (8)	Reduction [%]	Erythromycin (256)	Reduction [%]	Norfloxacin (4)	Reduction [%]
**PA**	**IJ26**	16	−100	8	96.9	0.5	87.5
	**IJ27**	8	0	128	50.0	1	75.0
	**IJ28**	8	0	128	50.0	0.62	84.5
	**IJ29**	16	−100	80.63	68.5	2	50.0

**Figure 1:**
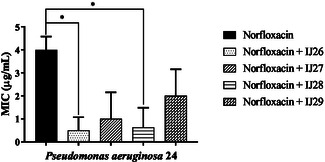
Modifying effect on norfloxacin action through association with thidiazines against *P. aeruginosa* 24. “*” *p *< 0.0314 indicates significant differences between groups. Statistical significance was determined by one‐way ANOVA and Bonferroni posthoc test.

To evaluate the interaction of the compounds with antibiotics, combination assays were performed. As shown in **Figure** **1**, compounds IJ26 and IJ28 significantly enhanced the activity of norfloxacin against *P. aeruginosa*, reducing its MIC from 4 μg mL^–1^ to 0.5 and 0.6 μg mL^–1^, respectively, representing a reduction of approximately eightfold and 6.65‐fold.

**Figure 2:**
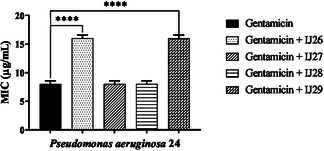
Modifying effect on the action of gentamicin through association with thidiazines against *P. aeruginosa* 24. “****” *p *< 0.0001 indicates significant differences between groups. Statistical significance was determined by one‐way ANOVA and Bonferroni posthoc test.

**Figure 3:**
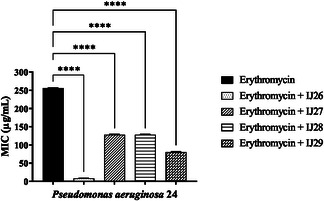
Modifying effect on the action of erythromycin through association with thidiazines against *P. aeruginosa* 24. “****” *p* < 0.0001 indicates significant differences between groups. Statistical significance was determined by one‐way ANOVA and Bonferroni posthoc.

**Figure** **2** shows the interactions between the compounds and gentamicin. Compounds IJ26 and IJ29 caused an increase in the MIC of the antibiotic, negatively affecting its mechanism of action. The MIC increased from 8 to 16 µg mL^–1^. Assays were also carried out to evaluate the interaction of the compound series in combination with erythromycin (**Figure** **3**). All compounds enhanced the bactericidal effect of erythromycin, with MIC values decreasing from 256 to 8, 128, 128, and 80 µg mL^–1^ when combined with IJ26, IJ27, IJ28, and IJ29, respectively.

**Figure 4:**
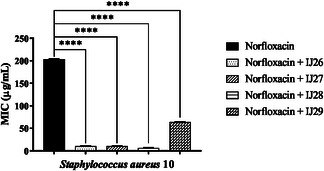
Modifying effect on norfloxacin action through association with thidiazines against *S. aureus* 10. “****” *p *< 0.0001 indicates significant differences between groups. Statistical significance was determined by one‐way ANOVA and Bonferroni posthoc.

Tests were conducted to evaluate the interactions of the compound series against *S. aureus* in combination with norfloxacin, gentamicin, and erythromycin (**Figure** **4**, **5**–**6**). Based on the results, all compounds combined with norfloxacin enhanced the antibiotic's effect.

**Figure 5:**
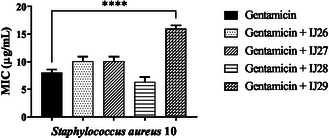
Modifying effect on the action of gentamicin through association with thidiazines against *S. aureus* 10. “****” *p* < 0.0001 indicates significant differences between groups. Statistical significance was determined by one‐way ANOVA and Bonferroni posthoc.

**Figure 6:**
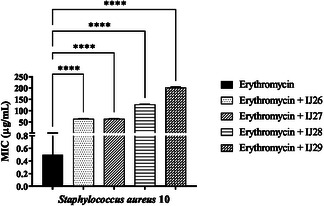
Modifying effect on erythromycin action through association with thidiazines against *S. aureus* 10. “****” *p* < 0.0001 indicates significant differences between groups. Statistical significance was determined by one‐way ANOVA and Bonferroni posthoc.

In combination with gentamicin, IJ29 caused an increase in the antibiotic's MIC, interfering with its mechanism of action, raising the MIC from 8 to 16 µg mL^–1^. When associated with erythromycin (Figure 6) against *S. aureus*, all compounds increased the MIC, which rose from 0.5 to 64, 64, 128, and 203 µg mL^–1^, respectively.

### 2.3 Virtual Screening of Target Prediction

A virtual screening model, which uses similarity testing with 3D/2D compounds from the ChEMBL database, constitutes a machine learning‐based strategy to estimate the therapeutic mechanisms of action of IJ26–29 analogs. It identifies both potential biochemical target classes (Supporting Information 3) and specific biological targets (Supporting Information 4).

The analysis revealed that ligands IJ26–28 predominantly interact with G‐protein coupled receptors (GPCRs), accounting for ≈30% of IJ26's predicted interactions. These include 5‐HT1A (286 similar compounds), 5‐HT2A (306), dopamine D2 (362), and muscarinic receptors M1 (94), M2 (68), M3 (109), and M5 (24). Twelve percent of IJ26's predicted targets are enzymes, including peripheral benzodiazepine receptors (183), serotonin transporters (81), acyl‐CoA cholesterol acyltransferase (96), desaturase (49), and the androgen receptor (70). Notably, IJ26 was the only analog predicted to interact with acetyl‐CoA carboxylase (51) (Supporting Information 4).

IJ28, with 24% of its targets among GPCRs, showed the highest similarity to opioid neuroreceptors, including kappa (126) and mu (200) receptors, and was the only analog predicted to interact with the dopamine D3 receptor (116).

Although IJ27 and IJ29 had fewer similar structures overall, they were predicted to interact with benzodiazepine receptors, serotonin transporters, and muscarinic M2 and M5 receptors. Moreover, IJ26 and IJ28 shared at least five similar 3D structures interacting with GABA‐A ion channels (subunits α1, α2, α3, α5) and at least 19 compounds similar to those binding acetylcholinesterase (AChE)—suggesting a mechanism of action involving both the central and peripheral nervous systems (Supporting Information 4).

### 2.4 Physicochemical Properties and Druglikeness

#### 2.4.1 Topological Analysis

From the molecular lipophilic potential (MLP) surface map, it was possible to observe the influence of substituent groups on ligand lipophilicity. Lipophilic substituents on IJ27 (4‐Cl) and IJ29 (4‐CH_3_) created two zones of strong hydrophobic contribution (blue in Supporting Information 5), yielding the highest partition coefficients (logP: 4.18 and 4.09, respectively). Their solubility indices (logS < −5.0) suggest these ligands are water‐soluble but retain internal nonpolar environments. In contrast, the addition of polar groups (O and OH) in IJ26 (4‐OCH_3_) and IJ28 (4‐OH) shifted logP below 4 and logS above −5.0, suggesting a better lipid–aqueous solubility balance.

#### 2.4.2 Ionization State by the pKa Calculation

Acid/base analysis indicated that the H‐bond donor in IJ28 renders it slightly acidic. The estimated pKa of 9.48 for the 4‐OH group implies ≈97.3% of the molecule is neutral at physiological pH (7.4), with ≈2.7% in the ionized form. All compounds contain the thiadiazine ring with a basic nitrogen (N3), showing low protonation (pKa = 3.35) at physiological pH (Supporting Information 5).

#### 2.4.3 Multiparameter Optimization (MPO)

The calculated physicochemical properties applied to the drug similarity criteria can be viewed in Supporting Information 6 and graphically in Supporting Information 7. With the results, it is possible to observe that the IJ26−29 analogs occupy a physicochemical space formed by compounds of high lipophilicity and slightly polar (logP > 3 and topological polar surface area (TPSA) ≤ 75 Å^2^) from the Pfizer, Inc. molecular database^[^ ^12^ ^]^ that are active in the central nervous system (CNS) (Supporting Information 7). In this predictive test, it is worth noting that the IJ28 ligand obtained the highest calculated topological polarity (TPSA = 56.98 Å^2^) with a strong contribution from the hydroxyl group of the substituted aromatic ring (4‐OH = 20.23 Å^2^), where the presence of 2 H‐bond donors (NH and OH) can decrease the effectiveness of permeability at the BBB.

From the golden triangle graph in Supporting Information 7, it is possible to observe that the most lipophilic compounds at physiological pH (logD at pH 7.4), that is, IJ27 and 29 (logD_pH 7.4_ > 4), move outwards of the spectrum that favors the oral absorption and metabolic stability of these ligands. On the contrary, the empirical decision showed that the IJ28 analog best fits within this physical–chemical space, where the logD_pH 7.4_ in the order of 3.26 (within the molecular weight (MW) range < 300 g mol^–^^1^) estimate that this compound can present, at the same time at the same time, high permeability and low liver destruction (Supporting Information 7).

Thus, the indications of MPO > 4.0 (on a scale between 0 and 6), for the IJ26−29 analogs, indicate that the alignment between their topological and physiological physicochemical properties favors the alignment between three fundamental pharmacokinetic attributes: high permeability passive, low susceptibility to P‐glycoprotein (P‐gp) efflux, and low metabolic clearance, including the possibility of activity in the CNS,^[^ ^13^ ^]^ constituting developed profiles of druglikeness (Supporting Information 6).

### 2.5 In Silico Absorption, Distribution, Metabolism, Excretion, and Toxicity (ADMET) Properties

#### 2.5.1 Evaluation of Pharmacokinetic Descriptors

In this study, it was possible to establish an explanation between drug similarity estimates and the in silico prediction of ADMET, where pharmacokinetic descriptors corroborate empirical decisions based on graph and molecular structure. The estimated P_app_ Caco‐2 values > 10 × 10^−6^ cm s^–^^1^ suggest that the compounds are highly permeable in intestinal cells, and when aligned with the low probability of the ligands being P‐gp substrates, lead to a human intestinal evaluation >80% (Supporting Information 8). Within the range of logCL < 1.0 mL min^−1^ kg^−1^, it is possible to note that the low hepatic clearance of the ligands guarantees an oral bioavailability of at least 30% (*F* 30%) in the systemic circulation.

Even so, the most lipophilic compounds, that is, IJ27 and 29, presented volume of distribution (VD) values > 0.2 Lkg, which indicates that they are more distributed in tissues than in blood plasma,^[^ ^14^ ^]^ contrary to which occurs in ligands IJ26 and 28 (Supporting Information 8).

#### 2.5.2 Site of Metabolism and Toxicity

Based on the structural analysis correlating ligand functional groups with hepatic metabolism sensitivity,^[^ ^15^ ^]^ the metabolic sites of analogs IJ26–29 were predicted (Supporting Information 7). The results indicate that electron‐donating groups, such as 4‐OCH_3_ (IJ26) and 4‐CH_3_ (IJ29), reduce the selectivity of cytochrome P450‐dependent aromatic hydroxylation, which may generate reactive epoxide intermediates. IJ26 undergoes O‐dealkylation, while IJ29 is metabolized by aliphatic hydroxylation through CYP2D6 and CYP2C9 pathways. In contrast, the less lipophilic IJ28 (logD = 3.26) shows higher resistance to phase I metabolism but forms glucuronide conjugates in phase II via UGT, whereas IJ27 is more susceptible to CYP2C9‐mediated aromatic hydroxylation (Supporting Information 7).

In this way, it was possible to observe that the resistance of the IJ28 ligand resulted in the lowest order of hepatic clearance (logCL = −0.06 mL min^−^^1^ kg^−^^1^), leading to a higher plasma concentration of the substance. On the contrary, metabolic processes do not result in severe damage to the human liver, resulting in a low probability of human hepatotoxicity (H‐HT) response (Supporting Information 8).

## 3 Discussion

The target class prediction method and the dataset used here constitute a virtual screening for biological targets based on 3D similarity testing with bioactive molecules from the ChEMBL structural library. This method differs from 2D similarity by overcoming structural reading interferences, such as molecular shape and electrostatic potential distribution.^[^ ^16^ ^]^ Recently, the target‐based virtual screening method using chemical structure similarity has enabled the understanding of the mechanism of action of new molecules, showing excellent correlation with in vitro and in vivo tests.^[^ ^17^ ^,^ ^18^ ^]^

For,^[^ ^19^ ^]^ from Pfizer, Inc., compounds with a weak basic center (hydrogen bond donors (HBD) < 1 with pKa of the most basic center < 8), as occurs in most commercially available medicines, which have high lipophilicity and low polarity (logP > 3 and TPSA ≤ 75 Å^2^) specific drugs with high permeability (P_app_ > 10 × 10^−6^ cm s^–1^), low passive efflux by intestinal P‐gp, low intrinsic clearance rate of the free molecular fraction in the HLM system and are active in S. However, small compounds (MW ≤ 500 g mol^–^^1^) with low moderate lipophilicity (logD_pH_ 7.4 ≤ 5) are leaders in optimizing oral absorption attributes and metabolic stability.^[^ ^20^ ^]^

Both topological physicochemical properties and physiological ones were applied to pharmaceutical criteria, where it was possible to establish a correlation between predictions based on empirical decisions and pharmacokinetic descriptors.

Predicting the metabolic sites of a substance allows us to avoid problematic compounds capable of forming reactive chemical products in hepatic metabolism. Biotransformations like aromatic hydroxylation, conducted by cytochrome P450 isoenzymes in the HLM, can create transition structures based on epoxides, chemically reactive and prone to causing hepatotoxicity.^[^ ^21^ ^]^ Therefore, substrates of various types of cytochrome P450 can lead to a pharmacokinetics‐based metabolism control of the administered oral dose.^[^ ^22^ ^]^

In microbiological and clinical assays, the MIC is commonly used to evaluate the effectiveness of new antimicrobials and the susceptibility of different strains. MIC values can be determined by various methods, such as disk diffusion, or more frequently, broth microdilution.^[^ ^23^ ^]^ Previous studies on synthetic thiadiazines reported strong antifungal activity but only moderate antibacterial effects, consistent with the present findings. However, most concentrations observed here lack clinical relevance due to the high amount of compound required relative to human blood volume.^[^ ^24^ ^]^

A study was carried out evaluating thidiazines against strains of *P. aeruginosa* 24 and *S. aureus* 10, and obtained a similar result in the present study, where thiadiazine derivatives performed better in the direct and potentiating activity of antibiotics against *S. aureus*.^[^ ^25^ ^]^

Studies carried out using thidiazines showed antibacterial, antifungal, antiviral, antiparasitic activity, among others. The scientific community has been researching the activity of thidiazine compounds against various microorganisms, and it has already been demonstrated to be a promising drug compound against Chagas disease.^[^ ^11^ ^]^

An analysis of synthetic thiadiazines showed efficacy against strains of *S. aureus*, *Bacillus subtilis*, *Klebsiella pneumoniae*, and Gram‐positive and Gram‐negative bacteria, respectively, MIC values were equal to or lower than standard drugs clotrimazole and chloramphenicol. The MIC value against *Candida albicans* and *Escherichia coli* was equal to standard medications.^[^ ^26^ ^]^

From the in silico ADMET test, it was possible to characterize a pharmacokinetic model where the IJ26−27 analogs, with high relative lipophilicity and low topological polarity, occupy a physicochemical space formed by CNS active drugs that are well absorbed and metabolized without toxic risk upon ingestion, acting as modulators of neuroreceptor activity.

The study demonstrated that thidiazine compounds potentiated the action of antibiotics. The compounds associated with norfloxacin against *S. aureus* showed greater significance in relation to the other medications. However, against *P. aeruginosa*, the association with erythromycin obtained more significant results. We can conclude that thidiazine analogs have antibacterial activity, however, more tests are needed to prove their mechanism of action and test these consequences in vivo.

## 4 Experimental Section

4.1

4.1.1

##### Virtual Screening of Target Prediction

The chemical structures of the IJ26–29 analogs were converted into simplified molecular input line entry system linear notations and subjected to virtual screening for biological target classes using the SwissTargetPrediction online server (http://www.swisstargetprediction.ch/). The tool was configured to perform a 3D similarity search with more than 370,000 bioactive compounds within a physicochemical space comprising over 3,000 macromolecular targets from the ChEMBL database, using the organism *Rattus norvegicus* as a reference. This process generated quantitative drug‐likeness predictions for the evaluated targets.

##### Virtual Screening of Target Prediction: Physicochemical Properties and Druglikeness

The 2D structures of the ligands were plotted using the academic version of Marvin 22.13.0 software (ChemAxon, https://chemaxon.com/products/marvin). This software was employed to calculate the MLP, logarithm of the partition coefficient (logP), distribution coefficient at physiological pH (logD_pH_ 7.4), MW, TPSA, HBD, hydrogen bond acceptors, and acid dissociation constant (pKa). These parameters were evaluated using Pfizer's 3/75 rule (logP ≤ 3 and TPSA > 75 Å^2^)^[^ ^12^ ^]^ and Pfizer's Golden Triangle rule (−2 < logD_pH_ 7.4 ≤ 5 and 200 < MW ≤ 500 g mol^–^^1^),^[^ ^20^ ^]^ both incorporated in predictive algorithm form on the ADMETlab 2.0 server (https://admetmesh.scbdd.com/).

The results were compared with the drug‐likeness score obtained from the MPO algorithm,^[^ ^13^ ^]^ a calculation plugin available on Marvin software. The MPO score ranges from 0 (poor) to 6 (optimal), based on six physicochemical criteria: logP ≤ 3, logD ≤ 2, MW ≤ 360 g mol^–^^1^, 40 < TPSA ≤ 90 Å^2^, HBD < 1, and pKa (most basic) < 8. Compounds with MPO scores > 4 were considered aligned with pharmacokinetic profiles associated with high permeability (P_app_), low hepatic clearance, and potential CNS activity.

##### Virtual Screening of Target Prediction: In Silico ADMET Properties

Following the methodology described by Rocha et al.^[^ ^27^ ^]^ two platforms were combined to generate a consensus prediction of the ADMET profile of the ligands. The platforms ADMETlab 2.0 (https://admetmesh.scbdd.com/) and pkCSM (https://biosig.lab.uq.edu.au/pkcsm/) were used to estimate the following parameters: apparent permeability (P_app_) in Caco‐2 cells, P‐gp substrate recognition, human intestinal absorption, oral bioavailability (F), VD, blood‐brain barrier permeability (logBB), and hepatic clearance (logCL). Metabolism was assessed using XenoSite (https://swami.wustl.edu/xenosite/submit), which predicted sites of metabolism based on structural inputs. These were compared with cytochrome P450 (CYP) inhibition predictions for isoforms CYP2C9, CYP2D6, and CYP3A4, as well as H‐HT, all predicted using ADMETlab 2.0.

##### Chemistry

All reagents and solvents were obtained from Sigma–Aldrich and used without further purification. Reaction progress was monitored by thin‐layer chromatography on Merck Kieselgel F254 silica plates under UV light (257 nm). Products were purified by silica gel (Merck 60, 0.040–0.063 mm) or flash column chromatography (Armen) using normal‐phase (Interchim 30 SHIP 25 g) or reverse‐phase C18 columns (AIT 50 g). ^1^H and ^13^C NMR spectra were recorded on a Bruker Avance III (600 and 150 MHz, respectively); chemical shifts (*δ*) and coupling constants (J, Hz) are reported with standard multiplicity designations (s, d, t, q, quint, br s, dd, td, m). Melting points were measured on a MSTecnopon PFMII apparatus (uncorrected). Compound purity was verified by high‐performance liquid chromatography (HPLC, Shimadzu SIL‐20AHT, Supelco Discovery C18 column) using methanol, methanol/formic acid, methanol/water, or water as mobile phases.

##### Chemistry: General Procedure for Synthesis

A mixture of thiosemicarbazide (1 mmol) and 2‐bromoacetophenone (1 equivalent) was dissolved in 15–20 mL of acetonitrile and stirred at room temperature for 1 h until complete consumption of the starting materials. The reaction mixture was then kept at room temperature, and the resulting solid was filtered. The final product was washed with methanol and water (Supporting Information 1 and 2).

##### 5‐(4‐methoxyphenyl)‐N‐phenyl‐6H‐1,3,4‐thiadiazin‐2‐amine (IJ‐26)

Yellow solid; yield 56%; mp 187–188 °C; HPLC‐UV: 7.84 min./95%; _1_H NMR (600 MHz, DMSO‐d_6_) *δ* 3.85 (s, _3_H, OCH_3_); 4.30 (s, _2_H, CH_2_); 7.12 (dt, _2_H, J = 3.10 e 8.95 Hz, H‐Ar); 7.34 (t, 1H, J = 7.38 Hz, H‐Ar); 7.45–7.50 (m, _4_H, H‐Ar); 7.92 (dt, _2_H, J = 3.10 e 8.95 Hz, H‐Ar); _13_C NMR (150 MHz, DMSO‐d_6_) *δ* 162.2; 154.4; 151.5; 137.7; 129.4; 129.2; 126.7; 123.9; 123.3; 114.5; 114.1; 55.5; 22.7.

##### 5‐(4‐chlorophenyl)‐N‐phenyl‐6H‐1,3,4‐thiadiazin‐2‐amine (IJ‐27)

Yellow solid; yield 60%; mp 179–180 °C; HPLC‐UV: 3.27 min./99%; _1_H NMR (600 MHz, DMSO‐d_6_) *δ* 3.74 (s, _2_H, CH_2_); 7.06 (dd, _2_H, J = 1.18 e 8.62 Hz, H‐Ar); 7,18 (tt, _1_H, J = 1.18 e 7.46 Hz, H‐Ar); 7.37 (dtq, _2_H, J = 1.95, 8.62 e 0.76 Hz, H‐Ar); 7,43 (dt, _2_H, J = 2.58 e 8.70 Hz, H‐Ar); 7.71 (dt, 2H, J = 2.58 e 8.70 Hz, H‐Ar); _13_C NMR (150 MHz, DMSO‐d_6_) *δ* 153.4; 145.9; 145.5; 136.1; 133.0; 129.1; 129.0; 127.4; 124.7; 122.4; 23.2.

##### 4‐(2‐(phenylamino)‐6H‐1,3,4‐thiadiazin‐5‐yl)phenol (IJ‐28)

Yellow solid; yield 60%; mp 111–112 °C; HPLC‐UV: 2.85 min./99%; 1H NMR (600 MHz, DMSO‐d_6_) *δ* 3.82 (s, _2_H, CH_2_); 6.82 (dt, _3_H, J = 2.86 e 8.86 Hz, H‐Ar); 7.01 (t, _1_H, J = 7.46 Hz, H‐Ar); 7.28 (dt, _3_H, J = 1.84 e 8.01 Hz, H‐Ar); 7.70 (s, _2_H, H‐Ar); 9.87 (s, _1_H, NH); 10.12 (s, _1_H, OH); _13_C NMR (150 MHz, DMSO‐d_6_) *δ* 159.3; 147.2; 129.1; 128.2; 126.4; 123.1; 122.0; 116.5; 115.9; 115.5; 22.6.

##### N‐phenyl‐5‐(p‐tolyl)‐6H‐1,3,4‐thiadiazin‐2‐amine (IJ‐29)

Yellow solid; yield 50%; mp 180‐181°C; HPLC‐UV: 3.18 min./99%; ^1^H NMR (600 MHz, DMSO‐d_6_) *δ* 2.39 (s, _3_H, CH_3_); 4.29 (s, _2_H, CH_2_); 7.35–7.38 (m, _3_H, H‐Ar); 7.42–7.43 (m, _2_H, H‐Ar); 7.50 (t, _2_H, J = 8.09 Hz, H‐Ar); 7.84 (d, _2_H, J = 8.3 Hz, H‐Ar); _13_C NMR (150 MHz, DMSO‐d_6_) *δ* 142.3; 130.6; 130.1; 130; 129.2; 127.7; 127.6; 124.8; 123.9; 23.1; 21.5.

##### Microorganism

To carry out the tests, strains of *P. aeruginosa* 24 and *S. aureus* 10 were used. These are Gram‐negative and Gram‐positive bacteria, respectively, and are part of MDR strains of their respective genera. All strains were maintained on brain heart infusion (BHI) agar, and prior to testing, they were incubated for 24 h at 37 °C in BHI agar. The resistance profiles of these strains can be found in the study by Bezerra et al.^[^ ^28^ ^]^

##### Microorganism: Preparation of Substances

Ten milligrams (10 mg) of each thiadiazine analog were weighed and stored in microtubes, then diluted in 0.5 mL of dimethyl sulfoxide (DMSO, Sigma–Aldrich). This solution was transferred to a 15 mL Falcon tube, and an additional 9265 µL of sterile distilled water was added, resulting in a final volume of 9765 µL with a concentration of 1024 µg mL^–1^. This solution was used for MIC testing and for evaluating the modulatory effect on antibiotic activity.

##### Microorganism: Preparation of Inocula and Antibiotics

On the day prior to testing, bacterial strains were cultured on heart infusion agar (HIA) medium in Petri dishes and incubated at 37 °C for 24 h to ensure proper growth. After incubation, a bacterial suspension was prepared by diluting colonies into sterile saline, in triplicate. The turbidity of the suspension was adjusted to match the 0.5 McFarland standard, equivalent to ≈1.5 × 10^8^ colony‐forming unit (CFU) mL^–1^. The antibiotics selected for testing were Gentamicin (aminoglycoside), Norfloxacin (fluoroquinolone), and Erythromycin (macrolide), all purchased from Sigma–Aldrich, at a stock concentration of 1024 μg mL^–1^.

##### Microorganism: Determination of MIC

MIC testing was performed in triplicate for each bacterial strain. Each microtube contained 1 mL of solution, composed of 900 μL of 10% liquid BHI medium and 100 μL of bacterial inoculum (10% of the total volume). Then, 100 μL of this inoculated medium was added to each well of a 96‐well microtiter plate, followed by a serial microdilution of 100 μL of the test substance. Final concentrations ranged from 512 to 8 μg mL^–1^, with the penultimate well serving as the microbial growth control.

The plates were incubated at 37 °C for 24 h, and after incubation, 20 μL of resazurin was added to each well. After 1 h at room temperature, results were recorded based on the colorimetric change from blue (resazurin) to pink (resorufin), indicating viable, metabolically active bacteria. Wells that remained blue indicated no detectable bacterial growth.

##### Microorganism: Antibiotic Action Modifying Activity

To assess the modulatory effect of the compounds on antibiotic activity, the methodology described by Coutinho et al.^[^ ^29^ ^]^ was followed. Thiadiazine solutions were diluted to 1024 μg mL^–1^, and each reaction tube contained BHI 10%, 150 μL of bacterial inoculum, and the test compound at subinhibitory concentration (MIC/8), resulting in 1.5 mL of total volume. For the control, tubes contained only BHI 10% and bacterial suspension. The 96‐well plates were filled by adding 100 μL of the test solution to each well, followed by serial microdilutions of 100 μL of the selected antibiotic, up to the penultimate well. The plates were then incubated at 37 °C for 24 h. Antibiotic concentrations tested ranged from 512 to 0.5 μg mL^–1^. The reading was performed as described in the MIC protocol, using resazurin as a viability indicator.

##### Statistical Analysis

The data were analyzed using one‐way ANOVA followed by post‐hoc tests. However, the assumptions of normality and homogeneity of variance were not formally tested in this study. We acknowledge this as a limitation and suggest that future investigations should include formal assessments to further strengthen the statistical analysis.

## Abbreviations

**Table:** 

CCCP	Carbonyl Cyanide 3‐Chlorophenylhydrazone
MAD	Meldrun´s Acid
EtBr	Ethidium Bromide
CPZ	Chlorpromazine
UFPR	Federal University of Paraná
MIC	Minimum Inhibitory Concentration
BHI	brain heart infusion
FIC	Fractioned Inhibitory Concentration
HIA	Heart Infusion Agar
CFU	colony‐forming unit
PBS	Phosphate Buffered Saline
ADME	Administration, Disponibility, metabolism and excretion
PDB	Protein Data Bank

## Conflict of Interest

The authors declare no conflict of interest.

## Supporting information

Supplementary Material

## Data Availability

The data that support the findings of this study are available from the corresponding author upon reasonable request.
