# Supplementary material for: Modifying Antibiotic Activity of Synthetic Thiadiazine Analogs Against MDR Bacteria and ADMET Analysis
Source: ChemistryOpen. 2025 Oct 24;15(4):e202500260. doi: 10.1002/open.202500260 (PMC13052028; doi:10.1002/open.202500260)
Supplement: Supplementary file 1 — Supplementary Material [file OPEN-15-e202500260-s001.pdf]

SM1. Synthetic route for preparing thidiazine derivatives. Reagents and conditions: (a) EtOH, room temperature, 12h, 95% (4). (b) MeCN, room temperature.

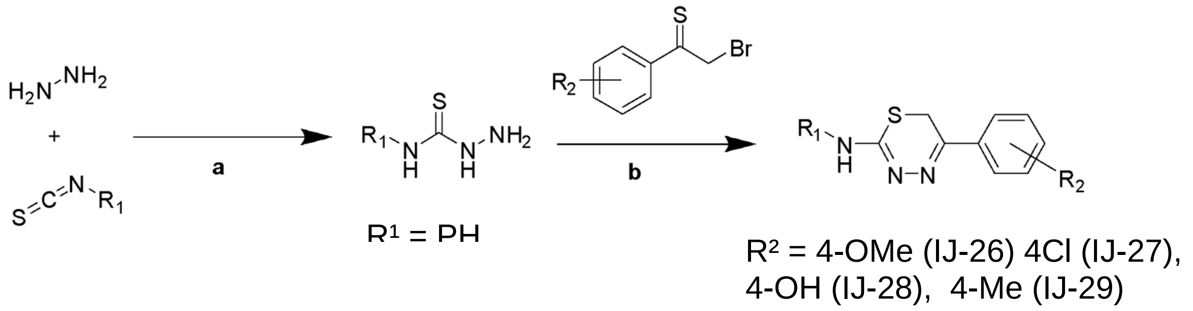

SM 2. Code of synthesized compounds and added radicals in the compounds.

Código	R1	R2
IJ-26	PH	4-OMe
IJ-27	PH	4Cl
IJ-28	PH	4-OH
IJ-29	PH	4-Me

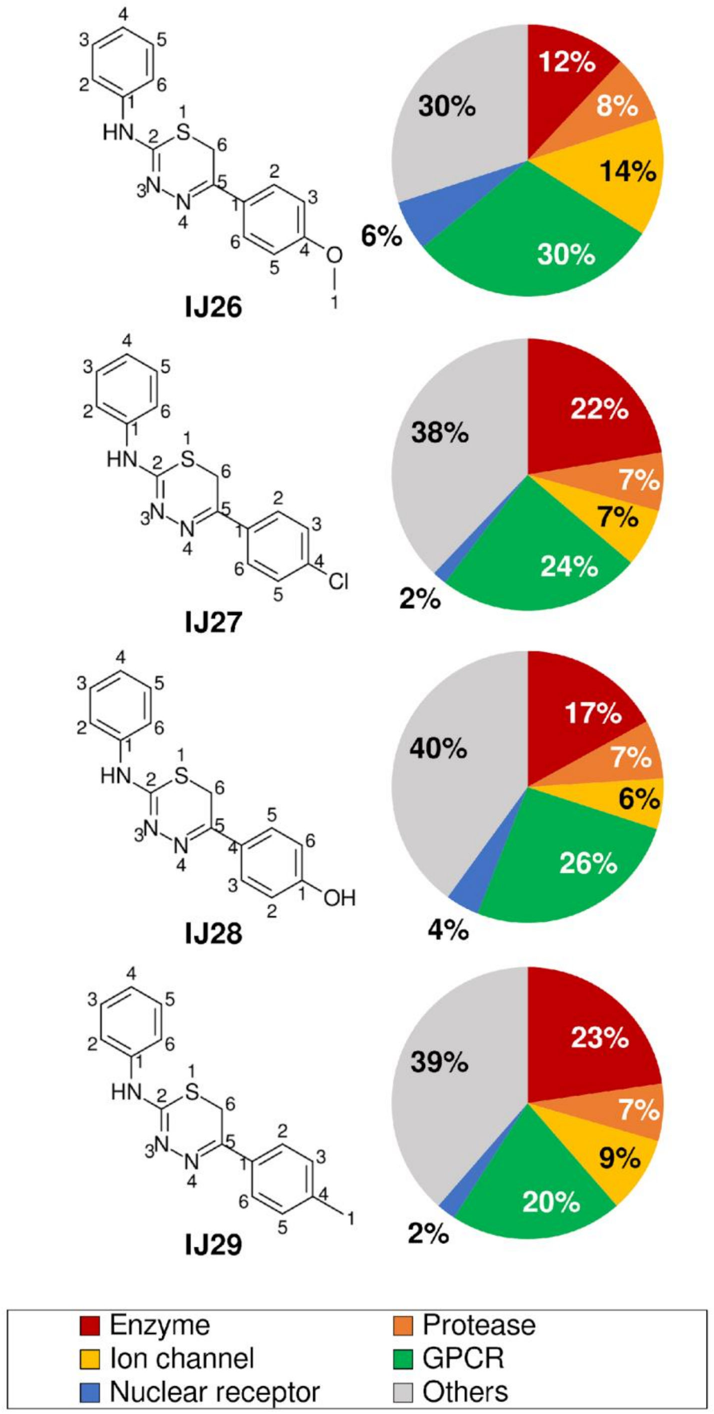

FIGURE 1 Supplementary Matrial 3. Virtual screening of target classes for the IJ26-29 analogues.

TABLE 1 Supplementary material 4. Results of virtual screening based on the identification of specific targets of biological activity by 3D similarity from the ChEMBL database.

**Target Name**	**Known actives similar (3D)**			
	**IJ26**	**IJ27**	**IJ28**	**IJ29**
Enzyme				
Acetyl-CoA: carboxylase 1	51	-	-	-
Acyl-CoA: cholesterol acyltransferase 1	96	4	46	3
Acyl-CoA: desaturase 1	49	-	34	-
Androgen Receptor	70	1	59	1
Aldehyde dehydrogenase 1A1	6	4	-	1
Peripheral-type benzodiazepine receptor	183	10	35	3
Serotonin transporter	81	-	90	-
G-Protein Coupled Receptors (GPCRs)				
5-HT 1a receptor	286	-	223	-
5-HT 1b receptor	-	-	32	-
5-HT 2a receptor	306	1	-	1
5-HT 2b receptor	10	-	-	-
5-HT 2c receptor	24	-	-	-
5-HT 3 receptor	17	-	12	-
5-HT 4 receptor	4	-	-	-
5-HT 7 receptor	9	-	-	-
Dopamine D2 receptor	362	2	392	-
Dopamine D3 receptor	-	-	116	-
Muscarinic acetylcholine receptor M1	94	-	39	-
Muscarinic acetylcholine receptor M2	68	2	38	2
Muscarinic acetylcholine receptor M3	109	1	61	-
Muscarinic acetylcholine receptor M5	24	2	-	1
Kappa opioid receptor	47	-	126	1
Mu opioid receptor	62	-	200	-
Monoamine oxidase A	89	-	44	1
Íon channel				
Monoamine oxidase B	144	-	50	1
GABA-A α1 receptor	18	-	11	-
GABA-A α2 receptor	11	-	10	-
GABA-A α3 receptor	5	-	5	-
GABA-A α5 receptor	56	-	11	-
Nuclear receptor				
Acetylcholinesterase	63	-	19	-

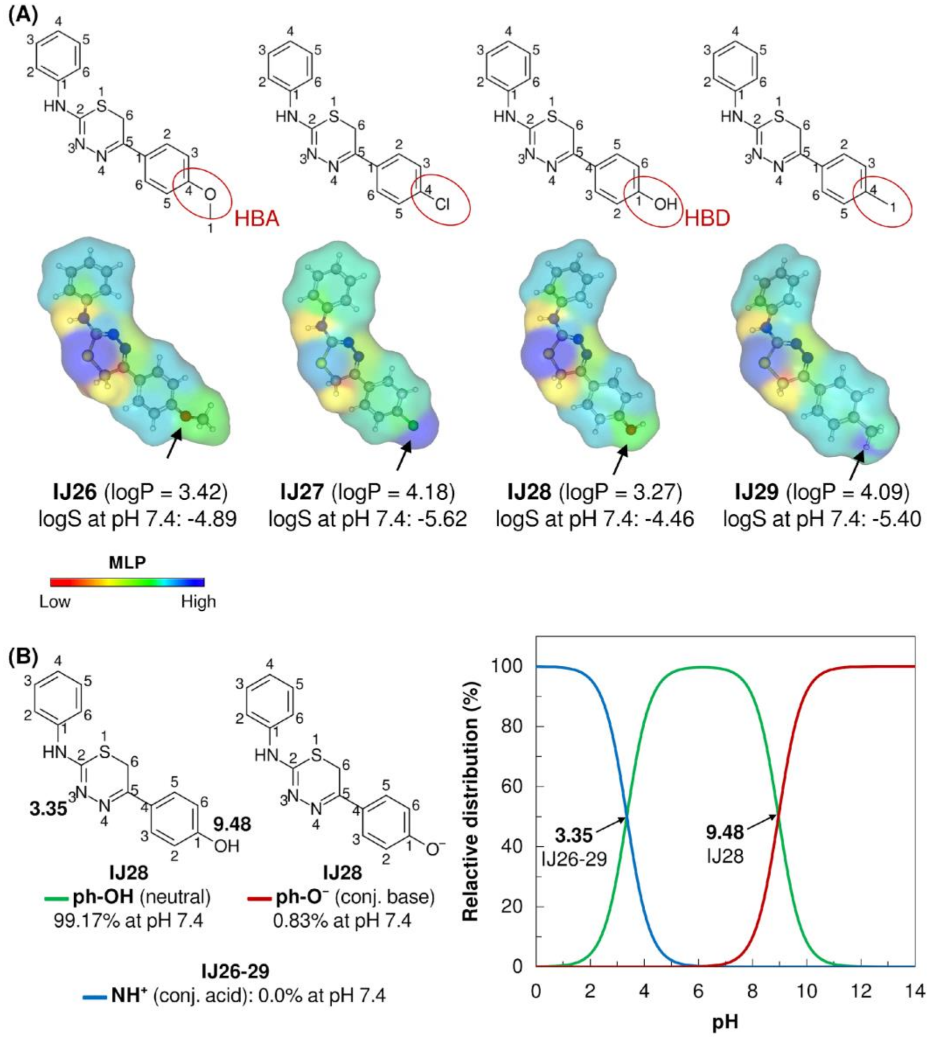

FIGURE 2 Supplementary material 5. Estimation of molecular lipophilic potential (MLP) (A) and Microspecies distribution graph as a function of pKa calculation (B).

Table 2. Physicochemical properties calculated for IJ26-29 analogues, applied to MPO druglikeness algorithm.

	IJ26	IJ27	IJ28	IJ29	Optimal
logP	3.42	4.18	3.27	4.09	≤ 3
$logD_{pH\ 7.4}$	3.42	4.18	3.26	4.09	≤ 2
MW	297.38 g/mol	301.79 g/mol	283.35 g/mol	281.38 g/mol	≤ 360 g/mol
TPSA	45.98 Å²	36.75 Å²	56.98 Å²	36.75 Å²	40-90 Å²
HBD	1	1	2	1	< 1
pKa (most basic)	3.35	3.35	3.35	3.35	≤ 8
Pfizer 3/75 rule	Rejected	Rejected	Rejected	Rejected	
Golden triangle	Accepted	Accepted	Accepted	Accepted	
MPO score	4.83	4.00	4.73	4.04	

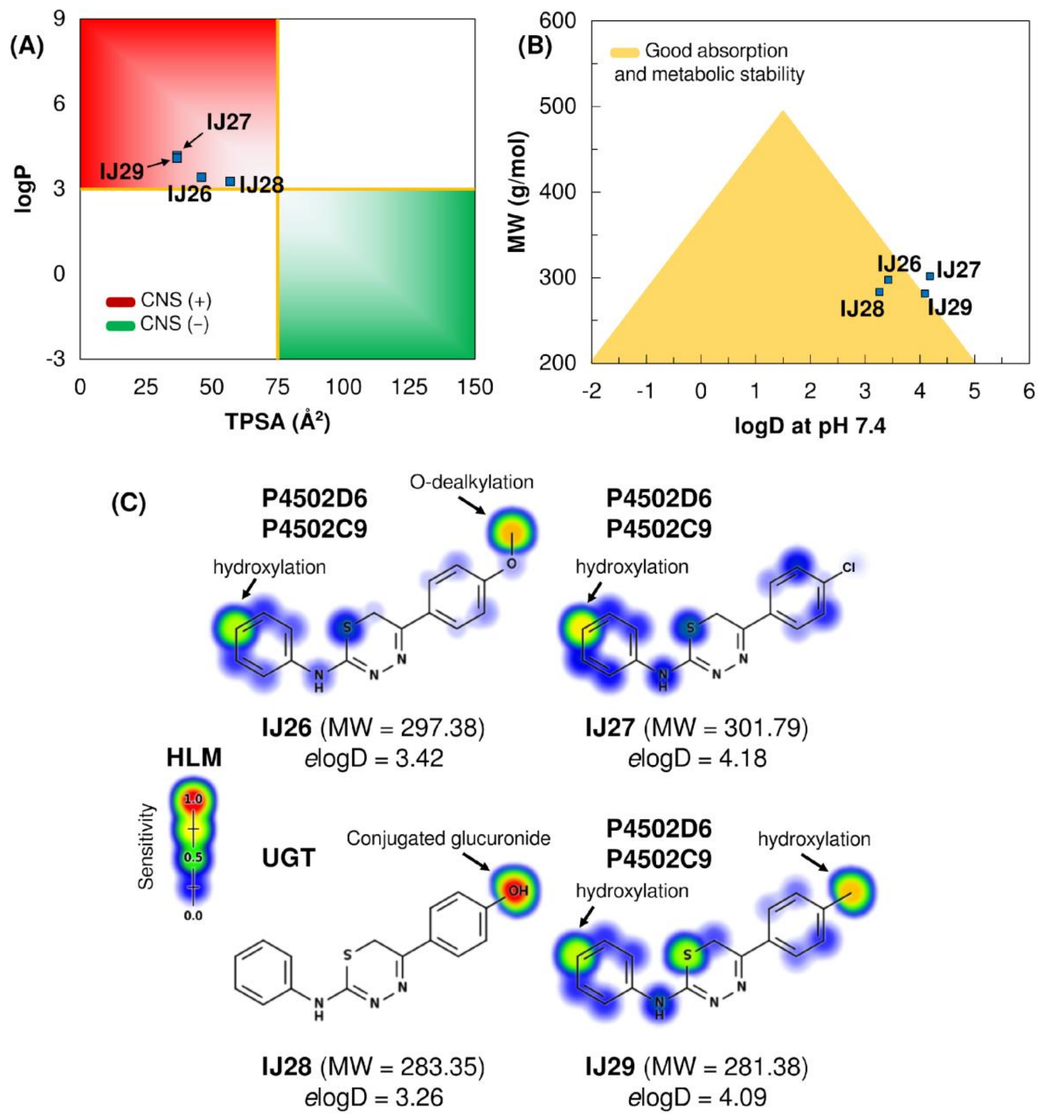

Figure 3. Pfizer 3/75 rule to estimate druglikeness for CNS activity (A), Golden Triangle rule to optimize permeability and metabolic clearance attributes (B) and structure-based site of metabolism prediction (C) for the IJ26-29 analogues.

Table 3. Pharmacokinetic descriptors estimated by consensus testing of ADMET models.

	**IJ26**	**IJ27**	**IJ28**	**IJ29**
Papp Caco-2b	$1.41x10^{-5}$ cm/s	$1.67x10^{-5}$ cm/s	$1.36x10^{-5}$ cm/s	$1.66x10^{-5}$ cm/s
P-gp substratea,b	---	---	---	---
HIAb	90.54%	88.72%	87.49%	90.17%
HIA<30%a	---	---	---	---
F<30%a	---	---	---	---
VDb	0.15 L/kg	0.27 L/kg	0.06 L/kg	0.31 L/kg
logBBb	0.32	0.44	0.21	0.46
P4502C9 inhibitora	+++	+++	++	+++
P4502D6 inhibitora	-	+	+	-
P4503A4 inhibitora	+	--	+	-
logCLb	0.08 mL/min/kg	0.04 mL/min/kg	-0.06 mL/min/kg	0.11 mL/min/kg
H-HTa	--	--	--	--

a: Predicted by the ADMETlab 2.0 web-tool

b: Predicted by the pkCSM web-tool

(---): Good result or low toxicity; (+++): Poor result or toxic.
